# Mycotoxin Detection through Colorimetric Immunoprobing with Gold Nanoparticle Antibody Conjugates

**DOI:** 10.3390/bios14100491

**Published:** 2024-10-10

**Authors:** Vinayak Sharma, Bilal Javed, Hugh J. Byrne, Furong Tian

**Affiliations:** 1 School of Food Science and Environmental Health, Technological University Dublin, D07 H6K8 Dublin, Ireland; 2 Nanolab, Physical to Life Sciences Research Hub, Technological University Dublin, D08 CKP1 Dublin, Ireland; bilal.javed@tudublin.ie (B.J.); hugh.byrne@tudublin.ie (H.J.B.)

**Keywords:** colorimetric sensor, bioconjugates, localized surface plasmon resonance, zearalenone, aflatoxin B1

## Abstract

Driven by their exceptional optical characteristics, robust chemical stability, and facile bioconjugation, gold nanoparticles (AuNPs) have emerged as a preferred material for detection and biosensing applications in scientific research. This study involves the development of a simple, rapid, and cost-effective colorimetric immuno-sensing probe to detect aflatoxin B1 and zearalenone using AuNP antibody (AuNP-mAb) conjugates. Anti-toxin antibodies were attached to the AuNPs by using the physical adsorption method. The colorimetric immunosensor developed operates on the principle that the optical properties of the AuNP are very sensitive to aggregation, which can be induced by a critical high salt concentration. Although the presence of antibodies on the AuNP surface inhibits the aggregation, these antibodies bind to the toxin with higher affinity, which leads to exposure of the surface of AuNPs and aggregation in a salt environment. The aggregation triggers a noticeable but variable alteration in color from red to purple and blueish gray, as a result of a red shift in the surface plasmon resonance band of the AuNPs. The extent of the shift is dependent on the toxin exposure dose and can be quantified using a calibration curve through UV–Visible–NIR spectroscopy. The limit of detection using this assay was determined to be as low as 0.15 ng/mL for both zearalenone and aflatoxin B1. The specificity of the prepared immunoprobe was analyzed for a particular mycotoxin in the presence of other mycotoxins. The developed immunoprobe was evaluated for real-world applicability using artificially spiked samples. This colorimetric immunoprobe based on localized surface plasmon resonance (LSPR) has a reduced detection limit compared to other immunoassays, a rapid readout, low cost, and facile fabrication.

## 1. Introduction

The demand for portable sensing technologies that can be employed in point-of-care diagnostics and field-testing applications in food industries is rapidly increasing. These technologies should be low-cost, rapid, and robust. Mycotoxins, the silent invaders in our food chain, are toxic compounds produced by certain types of fungi, posing a significant threat to the health of humans and animals worldwide [1]. Among other mycotoxins, aflatoxin B1 and zearalenone are of particular concern, as they can cause cancer, suppress the immune system, impair growth, and interfere with the reproductive system [2]. Aflatoxins (AFBs) are a type of mycotoxin produced by species of fungi, namely *Aspergillus flavus* and *Aspergillus parasiticus* [1]. Zearalenone (ZEN), also known as F-2 toxin, is similarly produced by members of the genus *Fusarium* such as *Fusarium graminearum*, *Fusarium oxysporum*, and *Fusarium nivalis* [3]. The European Commission has established a maximum limit of 5 µg/kg for AFB and 10 µg/kg for ZEN in raw cereal grain [4,5]. These mycotoxins pose a significant threat to food safety and human health worldwide; hence, their detection and quantification have become a field of paramount importance in ensuring the wellbeing of global communities.

The use of immunoprobes combined with colorimetric, fluorescent, or electrochemical transducers for food analysis applications has gained a lot of attention in recent years [6,7]. The colorimetric detection approach yields visible results that facilitate easy observation with the unaided eye and spectrometric quantification. Moreover, rapid advancements in nanotechnology have paved the way for the utilization of diverse nanomaterials in the fabrication of innovative biosensors. These immunoprobes are characterized by their simplicity, speed, cost-effectiveness, high sensitivity, and high selectivity in target detection, capitalizing on the unique properties of nanomaterials [8,9]. In particular, gold nanoparticles (AuNPs) possess several distinct characteristics that make them highly applicable in the field of sensing and detection: (i) AuNPs exhibit optical properties which reflect the shape, size, and interparticle distance [10]; (ii) well-dispersed AuNPs demonstrate a superior fluorescence quenching effect compared to their aggregated counterparts, a feature that has been extensively utilized in the development of sensitive fluorescent sensors for mycotoxin analysis [11]; (iii) the surface of AuNPs is characterized by a highly dense electron-rich layer, conferring dielectric and catalytic properties, as well as excellent biocompatibility [12]; (iv) AuNPs have good electrical conductivity and a macroscopic tunneling effect facilitating the rapid transmission of electrons to achieve signal amplification [13].

Studies have shown the effectiveness of AuNPs for their application in colorimetric detection, such as peptide-functionalized AuNPs for the colorimetric sensing of proteins [14]. Chen et al. reported a similar assay for enzyme detection, whereby the activity and concentration of a target analyte can be assessed with a very low limit of detection (LOD), ~5 nM [15]. AuNPs have also been employed for the colorimetric detection of different types of mycotoxins by antibody-functionalized AuNPs, including ochratoxin A, Aflatoxin B2, and fumonisin B1, yielding detection limits as low as 2 nM for fumonisin B1 [16]. An immunoassay based on a glucose peroxidase reaction was used for the detection of fumonisin in the concentration range 3–25 ng/mL [17].

Most of the AuNP-based immunoassays mentioned rely on the color change resulting from the aggregation of AuNPs. The basic principle behind the assay involves the change in the localized surface plasmon resonance (LSPR) of AuNPs. AuNPs have high molar extinction coefficients and show distinct size-dependent color changes [18]. The red color of AuNPs changes to purple and then blueish gray as a result of aggregation in the presence of the target analyte that can be observed and provides a simple and rapid method for the detection of a specific analyte.

**Figure 1:**
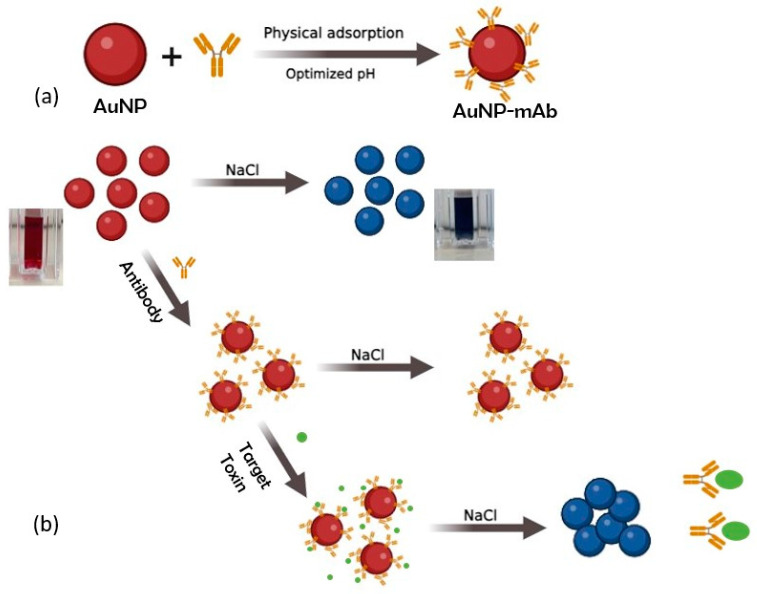
Schematic for (**a**) synthesis of gold nanoparticle (AuNP)—antibody (mAb) conjugate; (**b**) colorimetric immunoprobe for the detection of mycotoxins using AuNP-mAb.

This study exploits the aggregation phenomenon to create a AuNP-based colorimetric test for the detection of mycotoxins. The operational principle of the colorimetric immunoprobe for the detection of ZEN and AFB1 is shown in Figure 1. The AuNPs maintain their stability due to the force of electrostatic repulsion. However, when subjected to a high concentration of NaCl, the AuNPs undergo aggregation, leading to a change in the color of the nanoparticles, as depicted in Figure 1. In simpler terms, the introduction of a high salt concentration disrupts the stability of the solution, resulting in the aggregation of AuNPs and a subsequent color alteration. However, the aggregation of AuNPs is prevented in the presence of the antibody layer around the surface of AuNPs, bonded through electrostatic attraction, providing stability even in a high-salt-concentration solution. This is attributed to the mutual repulsion occurring between the negative charges present on the antibodies. Thus, the addition of a high concentration of NaCl to the AuNPs does not result in precipitation and the color of the solution remains red. Conversely, when the target analytes (ZEN and AFB1) are added to the reaction mixture containing AuNP-mAb conjugates, the antibodies detach from the AuNP surface due to the high-affinity interaction between the antibody and toxin [19]. This results in the loss of the protective effect on AuNPs, rendering them more prone to aggregation [20]. According to [21], these antibodies then bind strongly with the target analytes, forming a folded complex structure. This interaction exposes the AuNP surface and the addition of salt cations to the solution, which triggers the aggregation of the AuNPs. As a result, the color of the solution transitions from red to blue or gray, correlating with the quantity of target analytes. In essence, the alteration in the AuNPs’ state can be visually observed and the corresponding signals can be quantitatively analyzed using UV/Vis/NIR spectroscopy. In the current study, a critical salt concentration was determined which leads to aggregation of the AuNPs after exposure to the toxin of interest. The degree of aggregation is dependent on the degree of exposure of the initial AuNP-mAb solution to the mycotoxin, and therefore can be used to qualitatively and quantitatively determine the presence and concentration of ZEN and AFB1 in the sample. The sensitivity and specificity of the immunoprobe towards ZEN and AFB1 are determined by the choice of the color-changing indicator and the conditions under which the colorimetric detection is performed. This study provides high sensitivity and specificity towards the detection of these specific mycotoxins. This allows for rapid and visual detection which can be both qualitative and quantitative. The antibody-based assay provides high specificity and affinity for target antigens and can be adapted for a broad range of targets. The detection process is relatively simple and requires short time analysis. Ideally, this phenomenon could be applied to the rapid screening and detection of mycotoxins from food samples, which then may be subsequently confirmed by other analytical techniques where necessary.

## 2. Materials and Methods

### 2.1. Materials

Gold (III) chloride trihydrate (HAuCl_4_.3H_2_O), trisodium citrate (Na_3_C_6_H_5_O_7_), sodium hydroxide (NaOH), ethyl(dimethylaminopropyl)carbodiimide (EDC), and N-hydroxy succinimide (NHS) were procured from Sigma Aldrich, Ireland, as were the aflatoxin B1 and zearalenone toxins. The anti-aflatoxin B1 antibody and the mouse monoclonal antibody against zearalenone were provided by Abcam, Ireland. An Elix Reference Water Purification System from Millipore, Ireland, provided the deionized water (DI), which was necessary to produce the solutions used during the course of the study. None of these compounds was modified or purified beyond what was required for their intended use.

### 2.2. Bioconjugation of Gold Nanoparticles to Monoclonal Antibodies

The ZEN-mAb and AFB1-mAb AuNP conjugates were created via electrostatic adsorption [22] at the ideal labeling pH of the AuNPs. To prepare stable AuNP-mAb conjugates, the pH should be optimized for the isoelectric point of the protein, and for each antibody a different pH is required [23]. Initially, AuNP solutions of varying pH levels were prepared by adding 0.5, 1, 1.5, 2, 2.5, 4, 5, and 8 μL of 0.2 M solution of K_2_CO_3_ to different vials of 1 mL AuNP solutions. Subsequently, ZEN-mAb and AFB1-mAb, both with a volume of 2, 4, 8, 10, and 20 μL, were introduced to the AuNPs with the previously adjusted pH levels in different vials. The mixture was then subjected to mixing for a duration of 30 min. The binding time and NaCl incubation time were adopted from [24]. After that, 100 μL of 10% NaCl was added to the above conjugate solutions, mixed evenly, and kept for 10 min. The ideal pH was determined by adding the least amount of K_2_CO_3_ necessary to maintain the red color.

An environment with a high salt concentration was used to assess the effectiveness of the antibody adhesion to the gold nanoparticles. If the particle surface is not fully covered with antibodies, agglomeration and eventual precipitation of these particles occur in the presence of cations in the salt solution [25]. As a result, the color of the ZEN-mAb AuNP or AFB1-mAb AuNP solutions shifts from red (λ_max_ 520 nm) to blueish gray.

ZEN-mAb and AFB1-mAb AuNP conjugate solutions showed detectable chromatic changes after 10 min of addition of 100 μL of a 10 wt.% NaCl solution. The lowest concentration of ZEN-mAb/AFB1-mAb that successfully prevented AuNP aggregation while maintaining the characteristic red color of AuNP conjugate solutions was found to be the optimal concentration. Spectrophotometric analysis at 520 nm was used in conjunction with visual inspection to determine the ideal pH value and antibody volume for the ZEN-mAb GNP and AFB1-mAb AuNP solutions.

From the above optimization procedures, 5 μL of 0.2 M K_2_CO_3_ (pH 8.5) was chosen for ZEN-mAb AuNP and 4 μL was chosen for AFB1-mAb AuNP (pH 7.5) conjugates. After adding 10 μL of mAb solutions, the rest of the active surface not covered with antibodies was blocked by adding 100 μL of 10% BSA for 30 min. Finally, the reaction mixture was centrifuged for 15 min at 8000 rpm in order to remove the supernatant. The precipitate was dispersed in a buffer solution of PBS (1 mL, 0.01 M, pH 8.5) containing 0.1% bovine serum albumin (BSA) and 0.1% sucrose, which protects the secondary structure of proteins, and stored at 4 °C for further use.

### 2.3. Colorimetric Immunoprobe Preparation and Determination Protocol

The process of optimizing and preparing the AuNP–mAb complex for use in a colorimetric immunoprobe proceeded as follows. Initially, 150 μL of AuNP-mAb solution was dispensed into each well of 96-well transparent microplates. Each well was supplemented with 30 μL of toxin solution, followed by incubation at 240 rpm for a duration of 15 min. After that, 30 μL of the optimized NaCl solution was added and incubated for a further 10 min. The changes in the color of the solution were noted. A UV/Vis/NIR spectrophotometer was employed to acquire the absorption spectra within a wavelength range of 300–700 nm. The standard curve was plotted for different concentrations of mycotoxins ranging from 0 to 30 nM. After adding NaCl, the absorbance at 520 nm gradually decreased, and a red shift in absorbance to 630 nm was observed. The degree of aggregation of the AuNPs was measured using the absorbance ratio A_630_/A_520_. All the readings were performed in triplicate (n = 3), along with a blank to compare visually, and the error bars denote the standard deviation. The limit of detection was then calculated using a linear equation fitted [26] from the slope of the calibration curve, which is defined as LOD = 3*S.D./mean, where S.D. is the standard deviation of the readings obtained from measurements performed in triplicate [27].

### 2.4. Analysis of Spiked Samples for Detection of AFB1 and ZEN

The colorimetric sensor was applied for the detection of AFB1 and ZEN in artificially spiked samples. The sample buffer solutions were prepared in 0.01 M PBS buffer pH 7.4 with different quantities of ZEN and AFB1 (1, 5, and 10 ng mL^−1^) to verify the feasibility of this method. The sensor was prepared in the same manner as described in Section 2.3. The changes in the optical properties of the sample solutions were observed by the naked eye and UV/Vis/NIR spectroscopy. Finally, the absorbance was used to calculate the detected concentration from the equation of the standard curve and, along with it, the recovery rate of the samples were measured. The blank solution, with just AuNPs-mAb, was prepared without the addition of mycotoxin for visual comparison and absorbance readings. All the readings were performed in triplicate (n = 3) to verify the reproducibility of the results.

## 3. Results and Discussion

### 3.1. Optimization and Characterization of AuNP-mAb Conjugates (AuNP-ZEN mAb/AuNP-AFB1-mAb)

**Figure 2:**
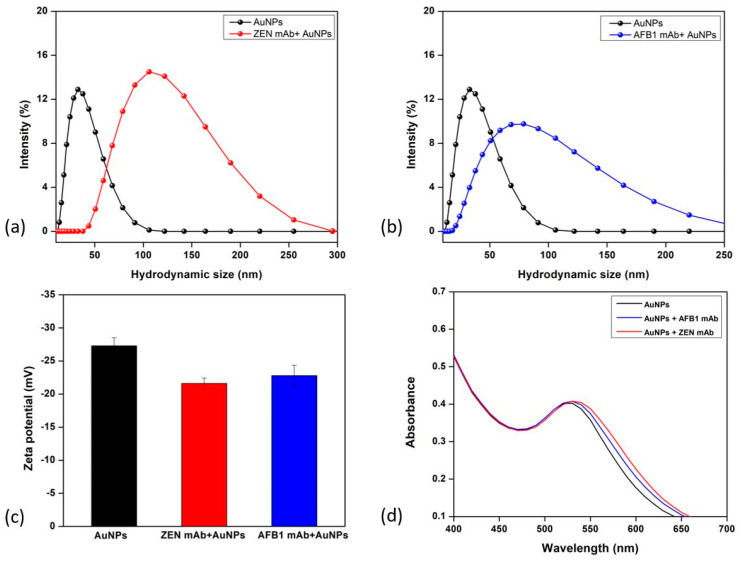
Gold nanoparticle-antibody conjugate characterization. (**a**) DLS size for bare AuNPs and anti-zearalenone attached antibody AuNPs; (**b**) DLS size for bare AuNPs and anti-aflatoxin B1 attached antibody AuNPs; (**c**) zeta potential analysis for bare AuNPs, ZEN mAb-AuNPs, and AFB1 mAb AuNPs; (**d**) change in UV-Vis spectroscopy after antibody attachment.

AuNPs of hydrodynamic diameter 33.3 ± 2.7 nm were prepared using a controlled chemical reduction of HAuCl_4_ with trisodium citrate, as described in Section 2. Supplementary Figure S1 depicts the hydrodynamic size of these AuNPs, as analyzed using DLS, and their morphology using scanning electron microscopy (SEM). Their UV/Vis/NIR absorption spectrum shows a characteristic absorption λ_max_ peak at 524 nm (Figure 2). The prepared AuNPs were successfully functionalized with anti-zearalenone or anti-aflatoxin antibodies through physical adsorption at pH 8.5 and 7.5. Different concentrations of antibodies were optimized within the pH range 6.5–9 in order to achieve a stable conjugate. At a lower pH of 6.5–7 and low antibody concentration, the color of the reaction solution changed from red to dark purple to grayish blue, indicating instability after NaCl was added. The selection of conditions for stable conjugates was conducted on the basis of a change in color and absorbance of the AuNP-mAb solution. The attachment of antibodies was confirmed by evaluating the change in UV/Vis/NIR absorption spectra, which depict the red shift from 524 nm to 529 nm, and the hydrodynamic diameter, as shown in Figure 2. This red shift occurs due to an alteration in the refractive index of the particle surroundings which was caused by the conjugation of antibodies on the biochemical corona layer on the particle surface [28]. Figure 2a,b show that the change in hydrodynamic size after the attachment of antibodies on the particle surface is confirmed by DLS. The AuNP diameter changed to 104 ± 2 nm in the case of ZEN mAb and to 82.3 ± 1.5 nm when the AFB1 mAb was conjugated. The zeta potential changed from −27 mV to −22.7 mV (AFB1 mAb) and −21 mV (ZEN mAb), indicating the stability of the conjugate formed in the solution (Figure 2c).

### 3.2. Detection of Zearalenone and Aflatoxin B1

**Figure 3:**
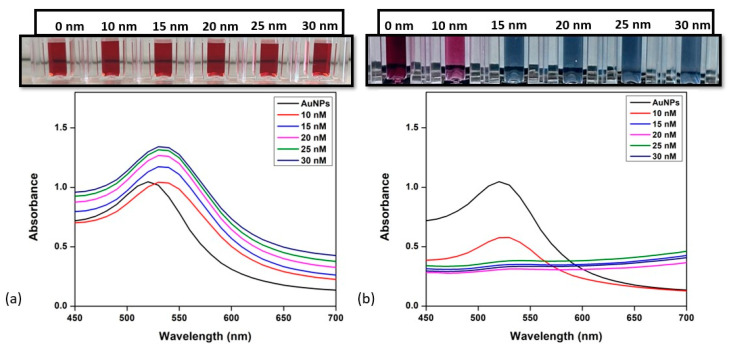
Detection of aflatoxin B1. (**a**) UV/Vis/NIR spectroscopy of AuNP-mAb after addition of different concentrations of aflatoxin; (**b**) change in UV/Vis/NIR spectra and color after inducing high salt concentration of 200 mM. Inset: visual color changes in corresponding solutions with added toxin concentrations.

**Figure 4:**
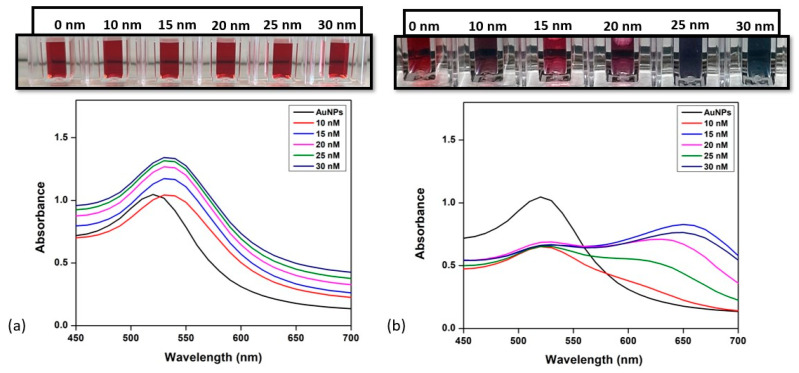
Detection of zearalenone. (**a**) UV/Vis/NIR spectroscopy of AuNP-mAb after the addition of different concentrations of zearalenone; (**b**) change in UV/Vis/NIR spectra and color after inducing high salt concentration of 200 mM. Inset: visual color changes in corresponding solutions with added toxin concentrations.

Under fine-tuned conditions of the concentration of antibodies and salt concentrations, the sensitivity of the colorimetric immunoprobe for the detection of zearalenone and aflatoxin B1 was examined. Various concentrations of ZEN and AFB1 were quantitatively identified using UV/Vis/NIR absorption spectroscopy as shown in Figure 3 and Figure 4. Both Figure 3 and Figure 4 depict the change in color and absorbance of the reaction mixture as a function of the concentration of toxin (aflatoxin and zearalenone, respectively) before and after the addition of NaCl. The addition of toxins to the AuNP-mAb solution results in the attachment of toxins to the antibodies present on the AuNP surface, which leads to a slight red shift in the UV/Vis/NIR absorption spectra, as shown in Figure 3a and Figure 4a. After incubating the reaction mixture, 200 mM of NaCl solution was added to each cuvette.

As the concentration of toxins is increased, the absorbance ratio (A_630_/A_520_ for AFB1 and ZEN) increases gradually after the addition of NaCl. For aflatoxin B1 detection, the increase in concentration from 10 nM to 30 nM resulted in a decrease in the absorbance peak at 520 nm, which shifted towards 630 nm, as seen in Figure 3. For the case of ZEN (Figure 4), the UV/Vis/NIR absorption spectra show the same red shift when the toxin is added, as it becomes attached to the antibodies present on the surface. On adding NaCl, the aggregation through color change was observed and a change in absorption peak at 630 nm and 530 was plotted. In both cases, the colorimetric assay was sensitive enough to visually detect results as low as 10 nM. The color of the reaction changed from red to dark pink due to the binding of the toxin to the antibody, exposing the surface of the AuNPs at 10 nM, and as the concentration increased and reached 30 nM, it changed to blueish gray, implying the aggregation of AuNPs in the presence of the toxin.

**Figure 5:**
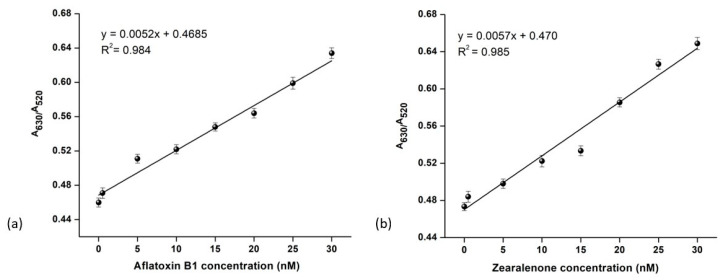
The sensitivity of the colorimetric sensor. Normalized absorbance values (A_630_/A_520_) of AuNP mAb-toxin solution versus the concentration of toxin ZEN (**a**,**b**) AFB1, respectively. All the readings were performed in triplicate (n = 3) and the error bars signify the standard deviation.

In Figure 5b, the absorbance values show a good linear correlation with the concentration of AFB1 from 10 to 30 nM, with the regression equation y = 0.0052x + 0.4685 and a correlation coefficient R^2^ = 0.984. The limit of detection was calculated to be 0.156 ng/mL, which is 0.5 nM (m.w. of AFB1—312.27 g/mol), lower than the maximum residue limit (5.0 µg/kg) as defined by the European Commission [5]. For ZEN, the sensitivity of the assay was assessed in the same range as AFB1. The absorbance ratio was seen to be linearly proportional to the concentration of ZEN in the range 10–30 nM (Figure 5a) and is fitted by the equation y = 0.0057x + 0.470, R^2^ = 0.985. The LOD of the assay was estimated to be 0.159 ng/mL, which is 0.5 nM (m.w. of ZEN—318.36 g/mol), lower than the maximum residue limit, which is 10.0 µg/kg [4].

A comparison of different detection methods of ZEN/AFBI is summarized in Table 1. In contrast to existing ZEN/AFB1 detection methods, this analytical approach offers several key benefits: it operates without the need for labeling, it is cost-effective, it involves straightforward sample preparation, avoiding complex procedures, and, most importantly, this method does not rely on expensive instrumentation.

### 3.3. Specificity of the Colorimetric Sensor

**Figure 6:**
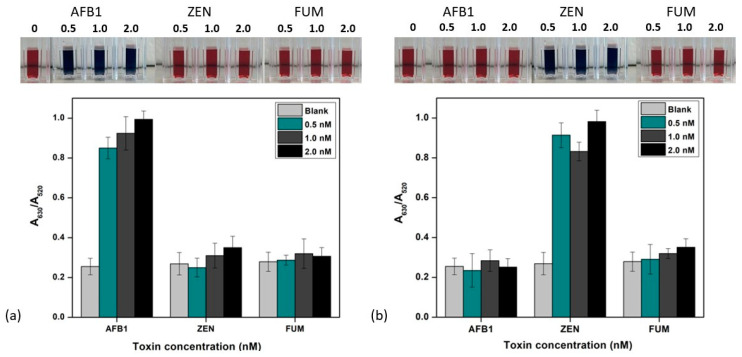
Selectivity of the colorimetric sensor for AFB1. (**a**,**b**) ZEN detection containing AuNP-mAb solution with different concentrations of toxins along with a blank (no mycotoxin). Inset: visual color changes in the corresponding solutions with added toxin concentrations. All the readings were performed in triplicate (n = 3).

To evaluate the selectivity and specificity of the developed colorimetric immunoprobe, various concentrations of different toxins not specific to the developed sensor were used, including fumonisin, aflatoxin, and zearalenone. These toxins were individually added to the colorimetric immunoprobe at concentrations of 0.5–2 nM and their response was investigated. The no-mycotoxin blank and the other concentrations were added to investigate the visual color change. As shown, there was no change in the color of the blank compared to other solutions and the absorbance ratio was the same. Figure 6a depicts the change in color which was observed when aflatoxin was added to the AFB-specific probe, while no significant change in color was observed when ZEN and FUM toxins were added at the same concentration. In the presence of the ZEN-specific probe, the color changed from red to blue and gray, but there was no change in the color in the presence of AFB1 and FUM toxins (Figure 6b). The absorbance ratio calculated A_630_/A_520_ in the presence of specific toxins was much higher than that of other toxins. The findings suggest that AFB1/ZEN uniquely induces a detectable signal change in the A_630_/A_520_ ratio, accompanied by a color shift from red to blueish gray [38]. In contrast, the colorimetric sensor remains relatively unchanged when exposed to other non-specific toxins under similar conditions. In summary, the developed immunoprobe exhibits high specificity, effectively avoiding interference from the other two toxins in the detection system.

### 3.4. Analysis of AFB1 and ZEN in Spiked Samples

To assess the utility of the colorimetric immunoprobe in various samples, the fabricated immunoprobe was utilized for the detection of ZEN and AFB1 in artificially contaminated samples. The sample buffer solutions were prepared in 0.01 M PBS buffer pH 7.4 with different quantities of ZEN and AFB1 (1, 5, and 10 ng mL^−1^), to verify the feasibility of this method. Table 2 shows the mean recovery rates of ZEN to be in the range from 93 to 101.4%, with relative standard deviation (RSD) from 0.57 to 5.53%, and 97.6 to 102.3% with an RSD from 4.18 to 11.64% in the case of AFB1. Note that the comparison of the recovery % and RSD in buffer was similarly measured in the reported studies [36,39]. The desirable recovery rates underscore the dependability of the devised method for the detection of AFB1 and ZEN. The results suggest that the proposed colorimetric immunoprobe possesses commendable feasibility and can serve as a viable alternative approach for the detection of mycotoxins in samples exhibiting high reliability.

## 4. Conclusions

In conclusion, a rapid and straightforward colorimetric assay utilizing gold nanoparticle–antibody conjugates for the detection of two mycotoxins using the corresponding antibodies has been engineered and characterized. The limit of detection was determined to be 0.15 ng mL^−1^ for both ZEN and AFB1. The reported assay is rapid and showed excellent specificity over other toxins, which makes it suitable for real-time monitoring and screening applications for mycotoxins. This type of method is a powerful tool, although it does have some limitations, including the limited spectrum range in using AuNPs, which means that it cannot detect substances beyond this range and can provide inaccurate results when dealing with complex mixtures with overlapping absorption spectra. An accurate bandwidth is required for more precise analysis. The proposed methodology, with continued refinement and in combination with other analytical methods, could emerge as a promising approach for the detection of mycotoxins, thereby contributing significantly to advancements in food safety.

## Figures and Tables

**Table 1:** Comparison of developed method in this work and other published methods.

Method	Material Used	Concentration Detected	Assay Time	Ref.
Lateral flow assay	AuNPs–antibody	ZEN—6 ng mL^−1^	15 min	[29]
Colorimetric	Fe_3_O_4_-GO	AFB1—5–250 ng mL^−1^	5 min	[30]
SERS (label-free)	AuNPs-PDMS@AAO	ZEN—47.7 ng mL^−1^	-	[31]
Lateral flow assay	AuNPs	AFB1—10 ng mL^−1^	15–20 min	[32]
Colorimetric	AuNPs–antibody	AFB1—12 ng mL^−1^	-	[33]
Colorimetric	AuNPs	ZEN—10 ng mL^−1^	>30 min	[26]
Colorimetric	AuNPs–antibody	ZEN—2.5 ng mL^−1^	-	[34]
Colorimetric	AuNPs–aptamer	AFB1—0.36 and 0.18 ng mL^−1^	<5 min	[35]
Colorimetric	AuNPS@ SiNPS-Ab	AFB1—0.16 ng mL^−1^	5–10 min	[36]
Colorimetric	AuNPs–aptamer	ZEN—5 ng mL^−1^	15 min	[37]
Colorimetric	AuNPs–antibody	AFB1 and ZEN—0.15 ng mL^−1^	<5 min	This work

**Table 2:** Test result of colorimetric sensor for AFB1/ZEN detection in spiked sample buffer solution (n = 3). (ND = Not detected, NC= Not calculated).

Compound	Spiked Concentration (ng mL^−1^)	Detected Concentration (ng mL^−1^)	Recovery (%)	RSD (%)
AFB1	0	ND	NC	NC
	1	0.97 ± 0.054	97.6	5.53
	5	5.1 ± 0.11	102.3	2.15
	10	9.98 ± 0.05	99.89	0.57
ZEN	0	ND	NC	NC
	1	1.01 ± 0.11	101.4	11.64
	5	4.65 ± 0.37	93	7.96
	10	10.05 ± 0.42	100.53	4.18

## Data Availability

Data are contained within the article.

## Supplementary Materials

The following supporting information can be downloaded at: https://www.mdpi.com/article/10.3390/bios14100491/s1, Figure S1. (a) Dynamic light scattering analysis of bare AuNPs, and salt induced aggregated AuNPs; (b) SEM of bare AuNPs; (c) Salt induced aggregation of AuNPs.

## References

[1] Klich M.A. (2007). *Aspergillus flavus*: The major producer of aflatoxin. Mol. Plant Pathol..

[2] Liu Y., Chang C.H., Marsh G.M., Wu F. (2012). Population attributable risk of aflatoxin-related liver cancer: Systematic review and meta-analysis. Eur. J. Cancer.

[3] Han X., Huangfu B., Xu T., Xu W., Asakiya C., Huang K., He X. (2022). Research Progress of Safety of Zearalenone: A Review. Toxins.

[4] Panel E.C., Schrenk D., Bignami M., Bodin L., Chipman J.K., Del Mazo J., Grasl-Kraupp B., Hogstrand C., Hoogenboom L.R., Leblanc J.C. (2020). Risk assessment of aflatoxins in food. EFSA J..

[5] Gallo P., Imbimbo S., Alvino S., Castellano V., Arace O., Soprano V., Esposito M., Serpe F.P., Sansone D. (2021). Contamination by aflatoxins b/g in food and commodities imported in southern italy from 2017 to 2020: A risk-based evaluation. Toxins.

[6] Chen H., Cai S., Luo J., Liu X., Ou L., Zhang Q., Liedberg B., Wang Y. (2024). Colorimetric biosensing assays based on gold nanoparticles functionalized/combined with non-antibody recognition elements. TrAC Trends Anal. Chem..

[7] Mahato K., Wang J. (2021). Electrochemical sensors: From the bench to the skin. Sens. Actuators B Chem..

[8] Malik S., Singh J., Goyat R., Saharan Y., Chaudhry V., Umar A., Ibrahim A.A., Akbar S., Ameen S., Baskoutas S. (2023). Nanomaterials-based biosensor and their applications: A review. Heliyon.

[9] Song F.-X., Xu X., Ding H., Yu L., Huang H., Hao J., Wu C., Liang R., Zhang S. (2023). Recent Progress in Nanomaterial-Based Biosensors and Theranostic Nanomedicine for Bladder Cancer. Biosensors.

[10] He Y., Tian F., Zhou J., Zhao Q., Fu R., Jiao B. (2020). Colorimetric aptasensor for ochratoxin A detection based on enzyme-induced gold nanoparticle aggregation. J. Hazard. Mater..

[11] Alhamoud Y., Yang D., Kenston S.S.F., Liu G., Liu L., Zhou H., Ahmed F., Zhao J. (2019). Advances in biosensors for the detection of ochratoxin A: Bio-receptors, nanomaterials, and their applications. Biosens. Bioelectron..

[12] Zeng R., Luo Z., Su L., Zhang L., Tang D., Niessner R., Knopp D. (2019). Palindromic Molecular Beacon Based Z-Scheme BiOCl-Au-CdS Photoelectrochemical Biodetection. Anal. Chem..

[13] Cai G., Yu Z., Ren R., Tang D. (2018). Exciton–Plasmon Interaction between AuNPs/Graphene Nanohybrids and CdS Quantum Dots/TiO_2_ for Photoelectrochemical Aptasensing of Prostate-Specific Antigen. ACS Sens..

[14] Aili D., Selegård R., Baltzer L., Enander K., Liedberg B. (2009). Colorimetric protein sensing by controlled assembly of gold nanoparticles functionalized with synthetic receptors. Small.

[15] Chen P., Selegård R., Aili D., Liedberg B. (2013). Peptide functionalized gold nanoparticles for colorimetric detection of matrilysin (MMP-7) activity. Nanoscale.

[16] Chen H., Zhou K., Zhao G. (2018). Gold nanoparticles: From synthesis, properties to their potential application as colorimetric sensors in food safety screening. Trends Food Sci. Technol..

[17] Chen X., Liang Y., Zhang W., Leng Y., Xiong Y. (2018). A colorimetric immunoassay based on glucose oxidase-induced AuNP aggregation for the detection of fumonisin B1. Talanta.

[18] Chang C.-C., Chen C.-P., Wu T.-H., Yang C.-H., Lin C.-W., Chen C.-Y. (2019). Gold nanoparticle-based colorimetric strategies for chemical and biological sensing applications. Nanomaterials.

[19] Wang A., Perera Y.R., Davidson M.B., Fitzkee N.C. (2016). Electrostatic Interactions and Protein Competition Reveal a Dynamic Surface in Gold Nanoparticle–Protein Adsorption. J. Phys. Chem. C.

[20] Filbrun S.L., Filbrun A.B., Lovato F.L., Oh S.H., Driskell E.A., Driskell J.D. (2017). Chemical modification of antibodies enables the formation of stable antibody–gold nanoparticle conjugates for biosensing. Analyst.

[21] Parolo C., de la Escosura-Muñiz A., Polo E., Grazú V., de la Fuente J.M., Merkoçi A. (2013). Design, preparation, and evaluation of a fixed-orientation antibody/gold-nanoparticle conjugate as an immunosensing label. ACS Appl. Mater. Interfaces.

[22] Busch R.T., Karim F., Weis J., Sun Y., Zhao C., Vasquez E.S. (2019). Optimization and Structural Stability of Gold Nanoparticle–Antibody Bioconjugates. ACS Omega.

[23] Ackerson C.J., Jadzinsky P.D., Jensen G.J., Kornberg R.D. (2006). Rigid, specific, and discrete gold nanoparticle/antibody conjugates. J. Am. Chem. Soc..

[24] Shahjahan T., Javed B., Sharma V., Tian F. (2023). pH and NaCl Optimisation to Improve the Stability of Gold and Silver Nanoparticles’ Anti-Zearalenone Antibody Conjugates for Immunochromatographic Assay. Methods Protoc..

[25] Okyem S., Awotunde O., Ogunlusi T., Riley M.B., Driskell J.D. (2021). Probing the Mechanism of Antibody-Triggered Aggregation of Gold Nanoparticles. Langmuir.

[26] Sun S., Zhao R., Feng S., Xie Y. (2018). Colorimetric zearalenone assay based on the use of an aptamer and of gold nanoparticles with peroxidase-like activity. Microchim. Acta.

[27] Şengül Ü. (2016). Comparing determination methods of detection and quantification limits for aflatoxin analysis in hazelnut. J. Food Drug Anal..

[28] Eck W., Craig G., Sigdel A., Ritter G., Old L.J., Tang L., Brennan M.F., Allen P.J., Mason M.D. (2008). PEGylated gold nanoparticles conjugated to monoclonal f19 antibodies as targeted labeling agents for human pancreatic carcinoma tissue. ACS Nano.

[29] Urusov A.E., Petrakova A.V., Zherdev A.V., Dzantiev B.B. (2016). “Multistage in one touch” design with a universal labelling conjugate for high-sensitive lateral flow immunoassays. Biosens. Bioelectron..

[30] Zhu W., Li L., Zhou Z., Yang X., Hao N., Guo Y., Wang K. (2020). A colorimetric biosensor for simultaneous ochratoxin A and aflatoxins B1 detection in agricultural products. Food Chem..

[31] Li J., Yan H., Tan X., Lu Z., Han H. (2019). Cauliflower-inspired 3d sers substrate for multiple mycotoxins detection. Anal. Chem..

[32] Yu S., He L., Yu F., Liu L., Qu C., Qu L., Liu J., Wu Y., Wu Y. (2018). A lateral flow assay for simultaneous detection of Deoxynivalenol, Fumonisin B1 and Aflatoxin B1. Toxicon.

[33] Wang X., Niessner R., Knopp D. (2014). Magnetic bead-based colorimetric immunoassay for aflatoxin b1 using gold nanoparticles. Sensors.

[34] Shim W.-B., Kim K.-Y., Chung D.-H. (2009). Development and validation of a gold nanoparticle immunochromatographic assay (icg) for the detection of zearalenone. J. Agric. Food Chem..

[35] Lerdsri J., Chananchana W., Upan J., Sridara T., Jakmunee J. (2020). Label-free colorimetric aptasensor for rapid detection of aflatoxin B1 by utilizing cationic perylene probe and localized surface plasmon resonance of gold nanoparticles. Sens. Actuators B Chem..

[36] Althagafi I.I., Ahmed S.A., El-Said W.A. (2021). Colorimetric aflatoxins immunoassay by using silica nanoparticles decorated with gold nanoparticles. Spectrochim. Acta Part A Mol. Biomol. Spectrosc..

[37] Zhang L., Chen J., Lu L., Yu R., Zhang D. (2023). A smartphone-assisted colorimetric aptasensor based on aptamer and gold nanoparticles for visual, fast and sensitive detection of ZEN in maize. Food Chem. X.

[38] Li R., Li L.Z., Huang T., Liu X., Chen Q., Jin G., Cao H. (2021). Gold nanoparticle-based colorimetric aptasensor for rapid detection of multiple mycotoxins in rice. Anal. Methods.

[39] Zhang W., Wang Y., Nan M., Li Y., Yun J., Wang Y., Bi Y. (2021). Novel colorimetric aptasensor based on unmodified gold nanoparticle and ssDNA for rapid and sensitive detection of T-2 toxin. Food Chem..

