# Supplementary material for: Mycotoxin Detection through Colorimetric Immunoprobing with Gold Nanoparticle Antibody Conjugates
Source: Biosensors (Basel). 2024 Oct 10;14(10):491. doi: 10.3390/bios14100491 (PMC11506043; doi:10.3390/bios14100491)
Supplement: Supplementary file 1 [file biosensors-14-00491-s001.zip › biosensors-3214113-supplementary.pdf]

Supplementary information

Mycotoxin detection through Colorimetric Immunoprobing with Gold Nanoparticle Antibody Conjugates.

Vinayak Sharma [1*], Bilal Javed [2], Hugh J. Byrne [2] and Furong Tian [2,*]

[1] School of food science and environmental health, Technological university of Dublin, Ireland; D21125324@mytudublin.ie
[2] Nanolab, Physical to life sciences research hub, Technological University Dublin, Ireland
* Correspondence: furong.tian@tudublin.ie

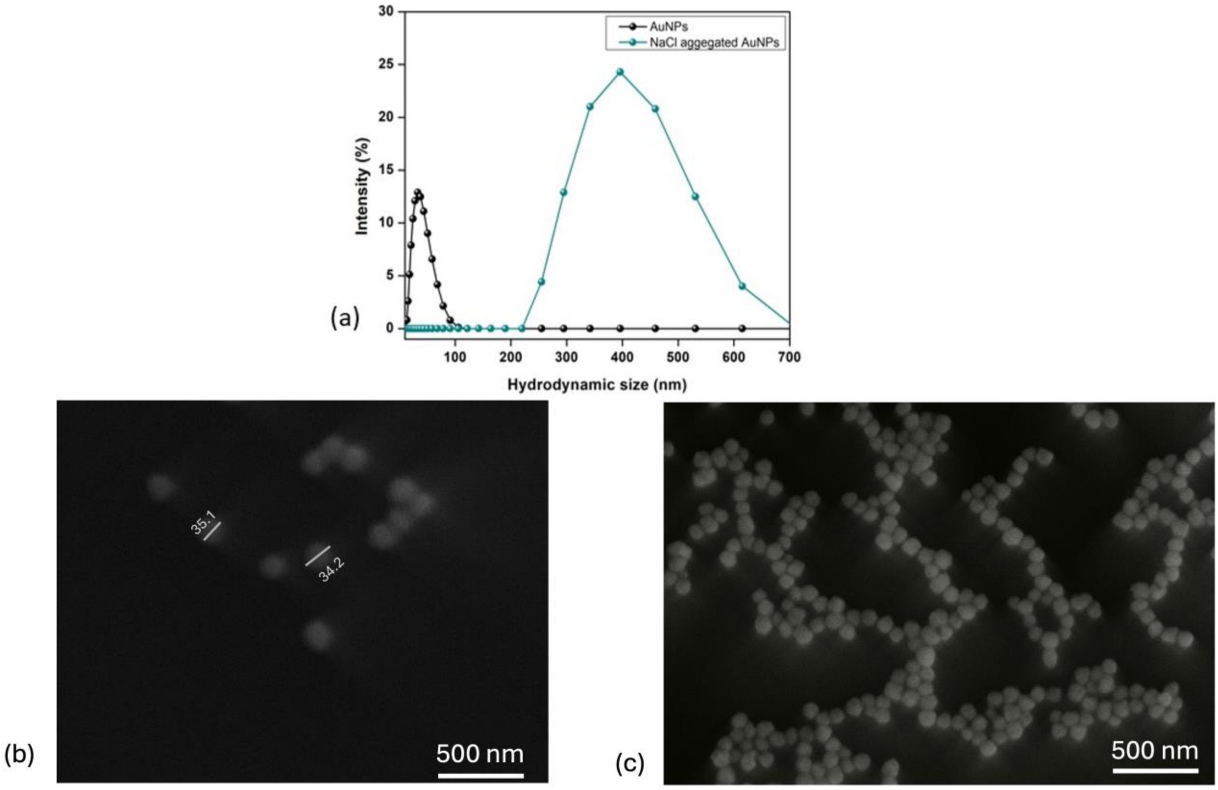

Supplementary Figure S1. (a) Dynamic light scattering analysis of bare AuNPs, and salt induced aggregated AuNPs; (b) SEM of bare AuNPs; (c) Salt induced aggregation of AuNPs.
